# Long-Term and Seasonal Trends in the Mode of Accumulation of Elements in the Bodies of Aquatic Insect Larvae

**DOI:** 10.1007/s00244-025-01144-7

**Published:** 2025-08-09

**Authors:** Martina Haas, Patrik Pánik

**Affiliations:** Institute of High Moutain Biology, University of Žilina, Tatranská Javorina 7, 05956 Tatranská Javorina, Slovak Republic

## Abstract

Aquatic insect larvae in high mountain streams reflect natural changes in water chemistry, making them effective bioindicators of ecosystem health. Changes in element accumulation in the bodies of larvae from the functional feeding groups scrapers and predators were monitored over a 5-year period (2019–2023). We aimed to evaluate long-term and seasonal trends in the accumulation of selected elements and to assess whether these patterns were linked to hydrological conditions or reflected post-disturbance recovery. Element concentrations were measured using ED-XRF spectrometry. Principal component analysis was used to identify accumulation patterns and their relationships with water flow rate and total dissolved solids (TDS). In scrapers, principal component (PC) 1 indicated general element accumulation, which varied significantly by year and season. PC2 showed increased in iron, copper, zinc, and lead with decreased in phosphorus and sulphur, with significant seasonal variation. PC3 linked an increase in iron and barium with higher flows and reduced chlorine and TDS, showing notable variation across years and seasons. In predators, PC1 also represented general element accumulation but involved fewer elements than scrapers. PC2 showed increased chromium and copper with higher flows and lower TDS, and PC3 indicated increased sulphur with higher flow and decreased zinc. Unlike scrapers, predators did not exhibit significant interannual variation, though seasonal variations were evident. These findings suggest that scrapers are more responsive to long-term environmental changes, while seasonal accumulation patterns in both groups are influenced by stream flow, food availability, and physiological changes during ontogeny.

**Supplementary Information:**

The online version contains supplementary material available at 10.1007/s00244-025-01144-7.

High mountain streams are unique ecosystems, and because they are little affected by human activities, they provide an important setting for research into and understanding of stream ecosystems and their functioning (Davis et al. 2001). Changes in the water chemistry of headwater streams depend on tectonics, geological structure, land cover, and land use (Allan and Flecker 1993; Yang et al. 2021), but also on the water regime of the landscape. The precipitation that feeds high mountain streams is a transport medium for contaminants originating from distant sources, which may adversely affect water quality in mountain streams. Aquatic insects are among the most directly affected and vulnerable organisms by surface water pollution and are an important component of biodiversity in lotic systems (Verneaux et al. 2003; Dikareva and Simon 2019).

Monitoring the physicochemical and biological properties of water is essential for understanding the biogeochemical cycles in mountain streams, as these cycles play a vital role in ecosystem functioning. Biogeochemical cycles involve the movement and transformation of nutrients and elements, which are influenced by ecosystem health. One effective method for assessing ecosystem health is the use of bioindicators, particularly aquatic insect larvae and macroinvertebrates, which serve as indicators of environmental quality (Mandaville 2002), due to their taxon-specific sensitivity to environmental conditions (Welti et al. 2024). In streams where fish populations are diminished due to pollution, macroinvertebrates can provide vital insights into ecological status (Rodriguez et al. 2018). Aquatic invertebrates function as intermediaries between producers and higher consumers in the food web, contribute to the early stages of biomagnification, and play a key role in ecological processes like nutrient cycling (Bano 2024), determining the impact of environmental pollutants such as heavy metals (Jeong et al. 2023).

As the concentrations of contaminants in the aquatic environment rise, shifts in aquatic insect populations may occur, with more tolerant species persisting under adverse conditions. This change can serve as an early warning of ecosystem degradation. Furthermore, the elemental concentrations found in the bodies of macrozoobenthos reflect the levels present in their surrounding environment (Kiffney and Clements 1993). Their distribution and sensitivity to stressors allow the detection of changes in the chemical and physical parameters of the environment (Chevalier et al. 2025). Trace elements, common contaminants in these ecosystems, can accumulate in aquatic organisms, providing a means to assess the availability of these elements and serving as indirect indicators of freshwater ecological health (Pastorino et al. 2019). Aquatic organisms can take up these trace elements directly from the water or through their diet, underscoring the interconnectedness of biotic and abiotic factors in aquatic ecosystems (Kanda 2024).

As part of trophic chains in ecosystems, aquatic insects fulfil functional roles ranging from detritivores to predators and provide a food source for vertebrates and invertebrates (Starr and Wallace 2021). The composition of macroinvertebrate functional feeding groups (FFGs) in lotic environments reflects key ecological processes. These organisms are categorised based on their feeding strategies and the type of organic matter they utilise. The primary FFGs include: scrapers which graze on periphyton and associated biofilms; shredders, which fragment coarse particulate organic matter such as leaf litter; collectors-gatherers, which exploit fine particles from benthic substrates; filter feeders, which extract suspended organic material from the water column, and predators, which consume other invertebrates. These classifications, originally proposed by Cummins and Klug (1979), have been refined in subsequent studies (Merritt and Cummins 2006; Ramirez and Gutiérrez-Fonseca 2014; Fierro et al. 2017; Doretto et al. 2020) and are widely used to assess ecosystem function and integrity. FFGs are not only ecologically distinct but also serve as effective tools in biomonitoring and bioindication. Because each FFG responds differently to environmental stressors, such as organic pollution, sedimentation, or metal contamination, their composition and abundance can reflect the ecological condition of aquatic habitats. For example, the dominance of collector-gatherers may indicate increased fine sediment or organic enrichment, while a decline in sensitive scrapers or shredders may signal habitat degradation or reduced water quality. As such, FFG-based assessments are widely used in ecological status evaluations and water quality monitoring frameworks (e.g., Bonada et al. 2006; Merritt et al. 2017). Collector-gatherer feeders, such as Oligochaeta, Diptera, and Chironomidae, are excellent accumulators (Pastorino et al. 2019), since they move into sediment and collect smaller particles of organic matter, thereby being exposed to metal-polluted substrates (Santoro et al. 2009).

In freshwater ecosystems, aquatic invertebrate taxa such as Ephemeroptera and Plecoptera are among the most abundant groups in mountain stream. These groups are extremely sensitive to environmental stressors and changes in the water quality, and they serve as reliable bioindicators of pristine conditions, requiring low pollutant levels, cool temperatures, and well-oxygenated water (Vilenica et al. 2022; Chowdhury et al. 2023). Their suitability as bioindicators is primarily linked to their larval stages, which are aquatic, relatively long-lived, and in direct contact with sediments and water, where they accumulate pollutants over time. Because adults are short-lived and often terrestrial, bioindication is based almost exclusively on larval traits and responses.

The life cycle of *Ecdyonurus venosus* (Ephemeroptera) includes distinct stages: egg, nymph, subimago, and imago (adult). The lifespan of the nymph can vary depending on the time of year in which eggs are laid. For example, most of the summer generations, and possibly some of the second summer broads, can complete their development in about a month. In contrast, most of the winter population and any offspring from a potential third brood spend 8 to 10 months overwintering as nymphs before completing their growth. The nymph stage undergoes numerous instars, with ten instars observed before reaching a length of 3 mm. Morphological changes between instars can be subtle, making it difficult to define instars based solely on morphological characters. (Rawlinson 1939).

The life cycle of *Perla grandis* (Plecoptera) is flexible in duration. Observations have shown that larval development cycles last 3 years in the Oriège River in the Pyrenees (Cereghino and Lavandier 1998) and four years in the Necker River in the Swiss Alps (Imhof 1994). Research also suggests the possibility of a 6-year life cycle in cold springs, based on models that link the length of embryogenesis to temperature (Bojková and Kroča 2011). These extended larval periods, combined with their sensitivity to environmental conditions, make Plecoptera particularly valuable for long-term biomonitoring.

The FFG framework also provides insight into the resilience of aquatic communities. Specialised feeders (e.g., shredders and scrapers) tend to be more vulnerable to habitat alteration and pollution, whereas generalists (e.g., collectors and filter feeders) exhibit greater tolerance due to their flexible feeding strategies (Barbour et al. 1996). Macroinvertebrates occupy intermediate trophic levels and mediate both bottom-up and top-down ecological interactions. Their activities influence nutrient cycling, organic matter decomposition, and energy transfer. Functional group-specific interactions with food resources further shape ecosystem dynamics (Wallace and Webster 1996). Elemental accumulation patterns vary among FFGs. Pastorino et al. (2020) reported that scrapers exhibited elevated concentrations of trace metals including Al, As, Bi, Co, Cd, Cr, Ga, Fe, In, Mn, Pb, Ni, and Sr). In contrast, predators showed higher levels of elements such as Ba, Hg, Li, Se, V, Ti, and Zn, while filter feeders accumulated Mo and Cu. Organisms that inhabit sediments and feed on detritus directly ingest metal-rich particles and exhibit a limited capacity to excrete certain trace elements. These differences likely reflect a combination of ecological exposure routes and physiological traits. Scrapers, which feed on periphyton, and biofilms attached to substrates, are in constant contact with sediment-bound metals and may ingest them along with their food. Predators accumulate metals primarily through trophic transfer, and their ability to metabolise or excrete certain elements depends on prey composition and metabolic efficiency. Filter feeders, by processing large volumes of water, are exposed to dissolved metals, which may explain their higher accumulation of waterborne elements (Pastorino et al. 2020). Thus, feeding strategy, habitat use, and physiological processing together shape the element-specific accumulation patterns observed among FFGs.

Our primary objective is to evaluate the long-term and seasonal trends in the accumulation of selected elements (P, S, Cl, K, Ca, Cr, Mn, Fe, Cu, Zn, Ba, and Pb) in larvae of two groups of benthic insects, classified according to their membership into the FFGs of scrapers and predators. We hypothesise that the rates of elemental accumulation will vary between the studied FFGs. The second main objective is to compare element accumulation across the study years to determine whether the observed patterns reflect cyclical changes or are influenced by external factors, such as rainfall variability in the region. The results will contribute to a better understanding of how elements are transferred within the aquatic environment across different seasons and years in each taxon. The research will offer insights into the natural accumulation of elements in selected aquatic insect assemblages in a stream that flows through an area with no direct anthropogenic influence that could cause pollution or contamination.

## Materials and Methods

### Characteristics of the Study Area

The research was conducted in the mountain stream Javorinka, located in the High Tatras (Western Carpathians, Slovakia). The Javorinka originates in the Javorová Valley, from the Žabie Javorové tarn (1,878.3 m a.s.l.), and has total length of 19.3 km. It flows through the Javorová Valley in the northeastern part of the Tatra Mountains, passing through the villages of Tatranská Javorina and Podspády, and eventually joins the Białka River on the Polish side. The Javorinka stream exhibits typical characteristics of alpine streams, such as low temperatures (0 to 10 °C), high flow variability (0.34 to 21.48 m^3^/s) with peak flows in late spring and early summer (late June) due to snowmelt, significant pH variability (4.7 to 8.4), and a heterogeneous vertical profile comprising cascades and step-pools with large boulders (SHMÚ - Slovak Hydrometeorological Institute 2018). The uppermost part of the basin is predominantly composed of granitoids and crystalline shales. In the lower sections, Mesozoic, Carboniferous, and Permian sedimentary rocks are mixed with alluvial sediments, influenced by the right-hand tributary, the Meďodolský stream, which originates in the limestone valley of Zadné Meďodoly.

**Fig. 1:**
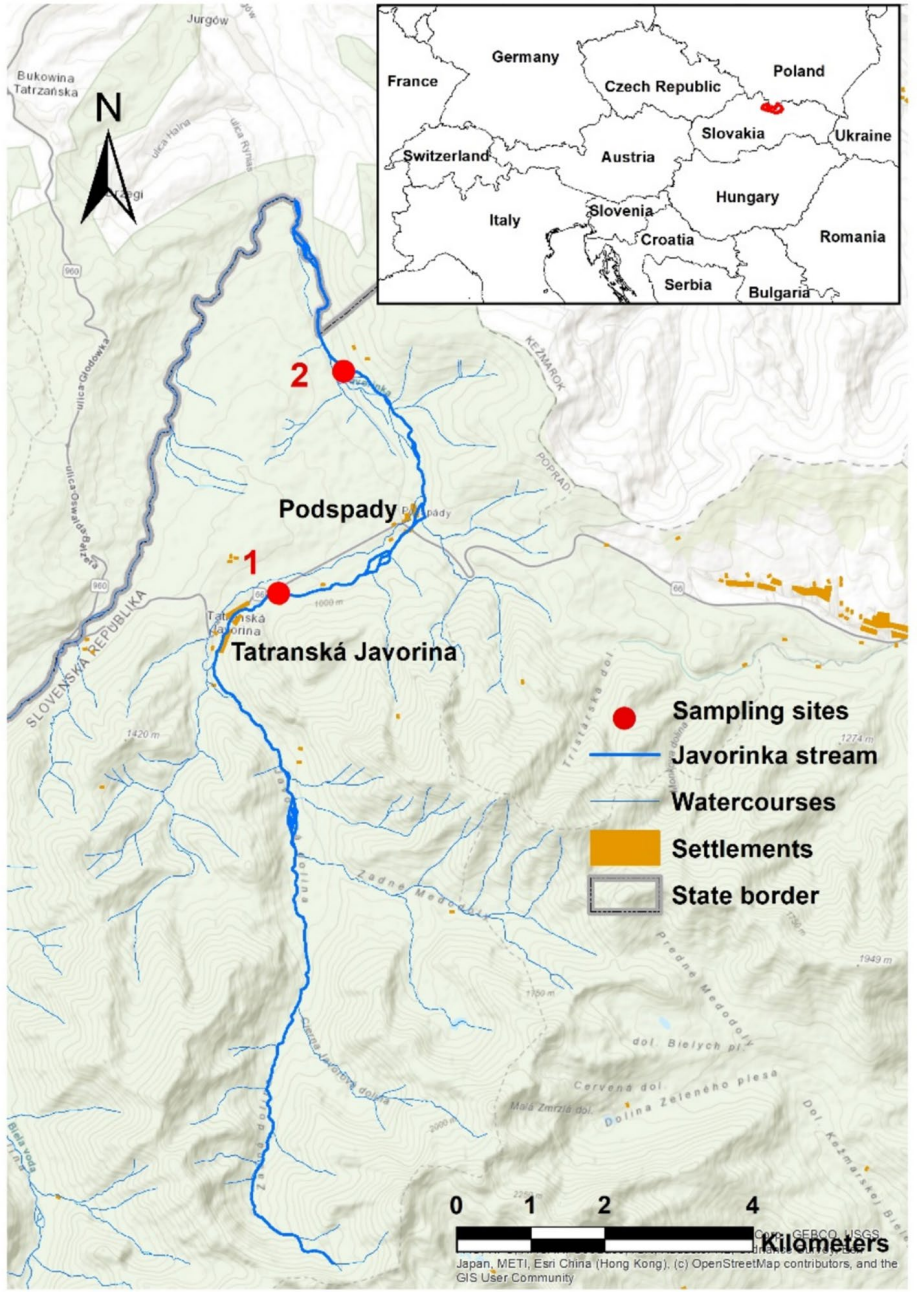
Location of two sampling sites in the lower river zone of Javorinka. The first site is situated near Tatranská Javorina (1), and the second is near Podspády (2). Data source: ESRI maps

To investigate the dynamics of elemental transport in the broader lower river zone, two sampling sites were selected (Fig. 1). The first site was located near the village of Tatranská Javorina (N 49°16´15.9; E 020°09´11.4; 962 m a.s.l.). The streambed in this section is primarily composed of larger rocks and gravel sediments along the stream margins. The second site was located near Podspády (N 49°17´52.4; E 020°09´40.9; 855 m a.s.l.). The rocks in this section of the stream are mainly granite, limestone, and shale, as the stream flows throught alternating bands of slate, flysch, and granite. The stream width at the sampling points ranges from 5 to 7 m.

The climate of the area is characterised as moderately cold to cold, with an average annual temperature of approximately 6 °C. The water flow rate varies seasonally. Average daily flow rates (m^3^.s^−1^) during the research period (2019–2023) were calculated using data from the Ždiar-Podspády station (Hydrological number 1–3-01–01–010–01) on the Javorinka watercourse, provided by Slovak Hydrometeorological Institute (SHMÚ), Regional Office Košice.

In winter, the flow is at its lowest, with calm water with temperatures around 3 °C. Spring is marked by snowmelt in late April and May, accompanied by rainfall, resulting in higher water level and temperature of 3–7 °C. Early summer brings the highest flow levels due to increased snowmelt and heavy rainfall. During late summer, water temperatures reach approximately 8–13 °C. In autumn, both flow and temperature (7–3 °C) begin to decline. Although rainy days still occur, their frequency is lower than in summer, and they are no longer associated with snowmelt. Both sides are situated far from the industrial zones, outside of heavily trafficked tourist areas, and lie between small villages with a combined population of approximately 150 inhabitants. The road runs parallel to the river at a distance ranging from a few metres to several tens of metres and includes a border crossing, which contributes to its relatively high traffic volume and frequent use by private vehicles. However, due to its location within the buffer zone of the Tatra National Park, the road is not treated with de-icing salts during the winter months.

### Sampling

Samples of aquatic insect larvae were collected regularly at the end of each month from spring (March to May) 2019 to autumn 2023 (September to November). All individuals were collected by direct hand-picking or with tweezers from rocks or from the streambed near the margins (to a depth of 20 cm), and placed in plastic bottles filled with stream water, separately for each group. Individuals were assigned to composite samples according to their classification within the functional feeding groups (FFGs): scrapers (Ephemeroptera, genus: *Ecdyonurus*, with *Ecdyonurus venosus* as the dominant species) and predators (Plecoptera, represented by a single species, *Perla grandis*). Each sample contained approximately 25–30 scraper individuals and 3–7 predator individuals, corresponding to approximately 0.5 g of dry weight.

Measurements of the physicochemical characteristics of the water were carried out at regular bi-weekly intervals as part of the institution’s independent research. Physical parameters (temperature, pH, total dissolved solids – TDS, dissolved oxygen, and water salinity) were measured in situ using a WTW 3430 portable multimeter (GEOTECH, Weilheim, Germany) equipped with compatible probes Multi 3430: IDS pH electrode Sen TixR 940–3; conductivity electrode TetraCon 925–3; optical oxygen electrode FDO 925–3. The average values of the physicochemical properties of the water during the study period are presented in Table 1.

**Table 1:** Average values of the physicochemical properties of the water during the study period, based on regular biweekly measurements

Variable	Valid N	Mean	Min	Max	SD	Unit
Temperature	370	5.63	0.10	13.10	3.25	°C
pH	371	8.21	7.00	8.86	0.23	
Concentration of O_2_	371	12.49	9.20	101.00	9.31	mg/L
O_2_	370	191.50	101.00	214.00	11.02	Mbar
Salinity	371	0.00	0.00	0.00	0.00	%
Total Dissolved Solids (TDS)	371	193.81	71.00	305.00	44.86	mg/L
Cl^−^	371	2.06	0.00	17.50	2.47	mg/L
Chlorides (CaCO_3_)	371	2.95	0.00	24.50	3.49	mg/L
NaCl	371	3.34	0.00	28.50	4.04	mg/L
Total hardness (CaCO_3_)	370	97.72	20.00	215.00	30.19	mg/L
SO_4_^2−^	371	10.84	0.00	67.00	11.19	mg/L
S	371	3.78	0.00	22.00	3.84	mg/L
Ammonia N	371	3.60	0.00	101.00	18.59	mg/L
Ammonia N-NH_3_	371	3.62	0.00	101.00	18.58	mg/L
Ammonia N-NH_4_	371	3.62	0.00	101.00	18.58	mg/L
Nitrates N	371	0.53	0.00	3.60	0.31	mg/L
Nitrates NO^3−^	371	2.31	0.00	4.47	0.95	mg/L
PO_4_^3−^	371	5.71	0.00	101.00	22.78	mg/L

(*N* number of samples; *SD* standard deviation)

### Laboratory Analysis

For each sampling event, individuals collected from the same site, date and FFGs were pooled into a single composite sample for analysis. All samples were air-dried at room temperature for an average of three days. The dried samples were manually crushed into fine powder using laboratory mortar and pestle to ensure optimal availability of elements for analysis. After homogenisation, the samples were analysed using an ED-XRF DELTA spectrometer (Innov-X Technologies, USA). This method provided a rapid and non-destructive approach and was well suited to the expected concentration ranges of the target elements. Despite its lower sensitivity compared to ICP-based methods, XRF spectrometry offers reliable relative elemental data, which are particularly useful when interpreted through multivariate techniques such as principal component analyse (PCA). As shown by Kumar et al. (2019), PCA applied to XRF data effectively reveals patterns of elemental distribution, making this approach suitable for ecological comparisons based on relative element proportions.

XRF spectrometry operates on the principle of irradiating the sample with high-energy X-rays, which excite the atoms and cause them to emit fluorescent X-rays. The emitted radiation is then detected and analysed to identify the elements present in the sample. The detector measures the fluorescent X-rays produced as atoms transition between energy levels, enabling both qualitative and quantitative analysis. Element concentrations are determined by measuring the intensity of their characteristic energy peaks. This methodology is widely used for quantitative identification of elemental composition across various materials in medicine, industry, and science. It enables the detection of elements such as: Cl, S, K, Ca, P, Rb, Zn, Mn, Mb, Fe, Ti, Sn, Co, Ni, Cu, As, Se, Pb, Sb, Ba, Hg, Cr, Ag, and Cd (in ppm). As there is no established standard for aquatic invertebrates, all samples were measured using the instrument´s default mode.

Each sample was measured for 120 s in triplicate, and an arithmetic mean of the element concentrations was calculated. The only elements consistently measured above the instrument´s detection limit (< LOD) were included in the analysis. Detection limits vary by element and were determined by the instrument manufacturer. These values are automatically calculated and displayed in the output matrix generated by the ED-XRF DELTA spectrometer software (Innov-X Systems 2018), based on internal calibration and signal-to-noise characteristics. The lowest detection limits were achieved using the Compton normalisation algorithm, which involves analysing a single, well-characterised standard and normalised the data to the Compton peak. Energy calibration control was performed after each instrument start-up or prior to measurement series, following the manufacturer's instructions. To assess analytical consistency and reliability, ten samples were randomly selected and subjected to ten replicate measurements. The relative standard deviation (RSD) was below 10%, indicating high precision.

Water chemistry parameters (chloride, nitrate, phosphorus, ammonia, sulphate, and total hardness) were determined using photometric analysis. Ion concentrations were measured with YSI EcoSense 9500 photometer (YSI Incorporated, USA), along with compatible accessories designed for this water analyser. Each chemical parameter required the use of specific reagents, as outlined in the test procedures. Measurements were conducted in accordance with the YSI EcoSense 9500 operation manual, using the optical analytical method.

### Statistical Analysis

Samples from both sampling sites were treated as a single dataset in the statistical evaluation, as both locations represent the lower course of the river. This approach was chosen to increase the number of observations per group and to enhance the robustness and interpretability of the results. Mean total element concentrations between the scraper and predator groups were compared using Student’s t-test (p < 0.05). To better understand the relationship between the accumulation of individual elements in the bodies of selected FFGs over the years, we applied principal component analysis (PCA), a multivariate statistical method. PCA is particularly suitable for assessing relationships among variables that are highly variable or measured in different units, as it reduces data dimensionality and reveals underlying patterns. Correlation analysis was also employed due to its greater sensitivity to outliers. As we assumed that the levels of each component differed among the FFGs being compared, these analyses were conducted separately for each group. Only the most abundant elements were included in this analysis. The most significant principal components (explaining more than 60% of the total variance) were further tested by one-way analysis of variance (ANOVA; 95% confidence level; p < 0.05) to determine differences in element accumulation patterns between years and within seasons across years. All statistical analyses were performed using Statistica, Version 12 (TIBCO Software Inc., USA).

## Results

The comparison of element concentrations between the functional groups scrapers and predators revealed statistically significant differences for certain elements. For example, the concentration of S was significantly higher in scrapers (8086.06 ± 4101.41 ppm) than in predators (6641.93 ± 3629.24 ppm; t = 2.48, *p* = 0.014), as were the concentrations of Mn, Fe, Zn, Ba, and Pb, all with *p* < 0.05 (Table 2). Only the concentration of Cl was significantly higher in predators (12,807.5 ± 6887.11 ppm; p = 0.0000). The average concentrations of individual elements within FFG groups, broken down by year and season, are provided in Supplement 1.

**Table 2:** Mean element concentrations (ppm) ± SD in the bodies of scraper and predator larvae

	Scrapers	SD	Predators	SD	t-value	*p*
P	2115.00	1406.42	2145.23	1274.50	− 0.15039	0.8800
S	**8086.06**	**4101.41**	**6641.93**	**3629.24**	**2.48403**	**0.0140**
Cl	**9652.15**	**4296.06**	**12,807.5**	**6887.11**	− **3.87831**	**0.0000**
K	25,219.80	9862.50	25,498.72	10,817.80	− 0.18332	0.8540
Ca	11,887.35	9320.98	12,018.36	7382.87	− 0.10273	0.9180
Cr	68.20	56.47	74.82	114.08	− 0.52829	0.5980
Mn	**239.30**	**83.36**	**175.11**	**95.59**	**4.89125**	**0.0000**
Fe	**2725.77**	**1952.47**	**1420.94**	**1624.76**	**4.81137**	**0.0000**
Cu	77.58	32.95	90.52	83.79	− 1.48055	0.1400
Zn	**520.53**	**246.68**	**431.43**	**163.68**	**2.76486**	**0.0010**
Ba	**143.76**	**49.20**	**121.65**	**61.07**	**2.74611**	**0.0010**
Pb	**13.97**	**4.52**	**12.60**	**2.88**	**2.34536**	**0.0200**

The results of the t-test (t-value) indicate the statistical significance of differences (*p* < 0.05 are highlighted)

### Element Accumulation in FFG Scrapers by Multivariate Comparison

**Fig. 2:**
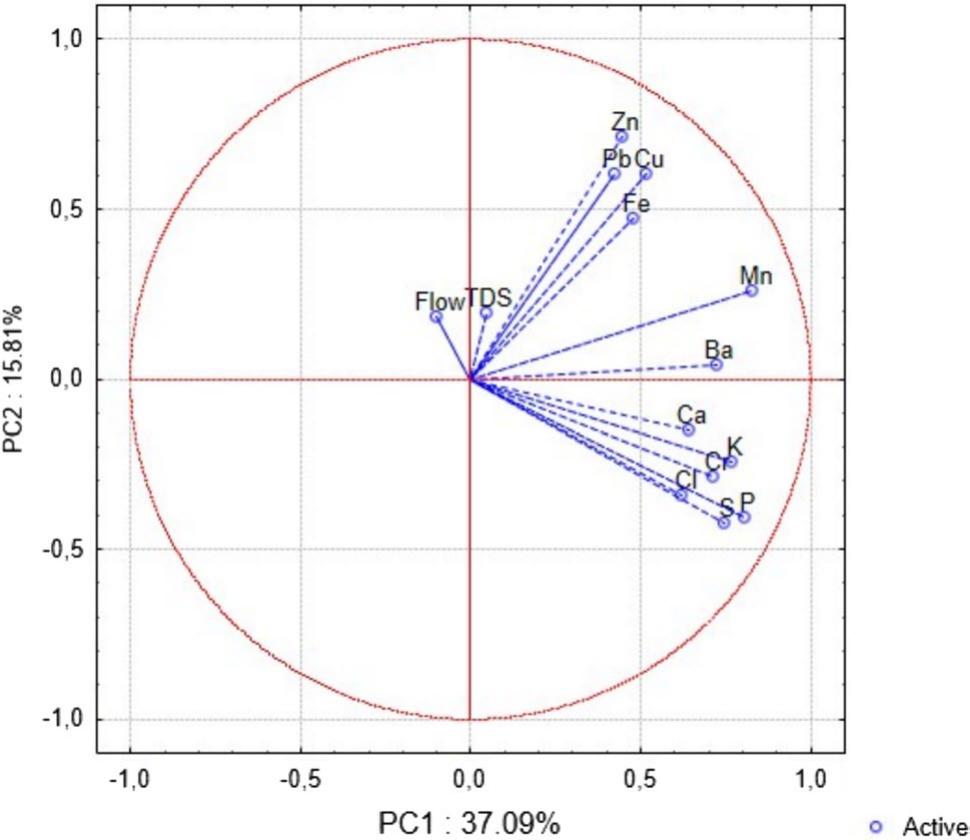
Biplot of principal component analysis for FFG scrapers, showing the distribution of active variables along the first two principal components (PC1 and PC2), which explain 37.09% and 15.81% of the total variance, respectively. Vectors represent the direction and strength of each variable's contribution to the principal components. Elements such as Zn, Pb, Cu, Fe, Mn, and others are projected in relation to water flow and TDS. The angle and length of the vectors indicate the correlation and influence of each variable on the PCA axes. Blue circles denote active variables included in the analysis

The interrelationship among the elements characterised by the main factors is significant in the first three principal components (PCs) within the scrapers functional feedings group (FFG) (Table 3; Fig. 2). These components explain more than 60% of the total variability of the dataset. PC1 represents the common accumulation trend of all elements (P, S, Cl, K, Ca, Cr, Mn, Fe, Cu, Zn, Ba, and Pb), while total dissolved solids (TDS) and flow rate do not contribute to this component. This suggests that the levels of these elements in the bodies of scrapers (Ephemeroptera) either increase or decrease in a uniform direction. PC2 reflects an increasing accumulation of Fe, Cu, Zn, and Pb, accompanied by a simultaneous decrease in P and S. PC3 indicates increased accumulation of Fe and Ba associated with higher flow rates, and concurrent decreasing Cl levels linked to lower TDS.

**Table 3:** Principal component (PC) coordinates of variables with the percentage of variation in the PCA for the most frequently and consistently detected elements in the bodies of invertebrate FFG scrapers, water flow rates, and TDS

	PC1	PC2	PC3
P	**0.805921**	− **0.405167**	− 0.011962
S	**0.745577**	− **0.423303**	− 0.030153
Cl	**0.621354**	− 0.341089	− **0.490271**
K	**0.767831**	− 0.241011	− 0.364630
Ca	**0.640393**	− 0.148046	0.342676
Cr	**0.712576**	− 0.287299	0.146014
Mn	**0.829457**	0.258300	0.084779
Fe	**0.479877**	**0.471591**	**0.441485**
Cu	**0.516536**	**0.601047**	− 0.360350
Zn	**0.442587**	**0.711678**	− 0.330849
Ba	**0.722738**	0.038819	0.528374
Pb	**0.422047**	**0.601858**	0.059591
Flow rate	− 0.100828	0.181177	**0.420030**
TDS	0.045396	0.195252	− **0.463833**
Total variance (%)	37.09	15.81	11.63

The coordinates that contribute the most to the PC value are highlighted in bold

**Fig. 3:**
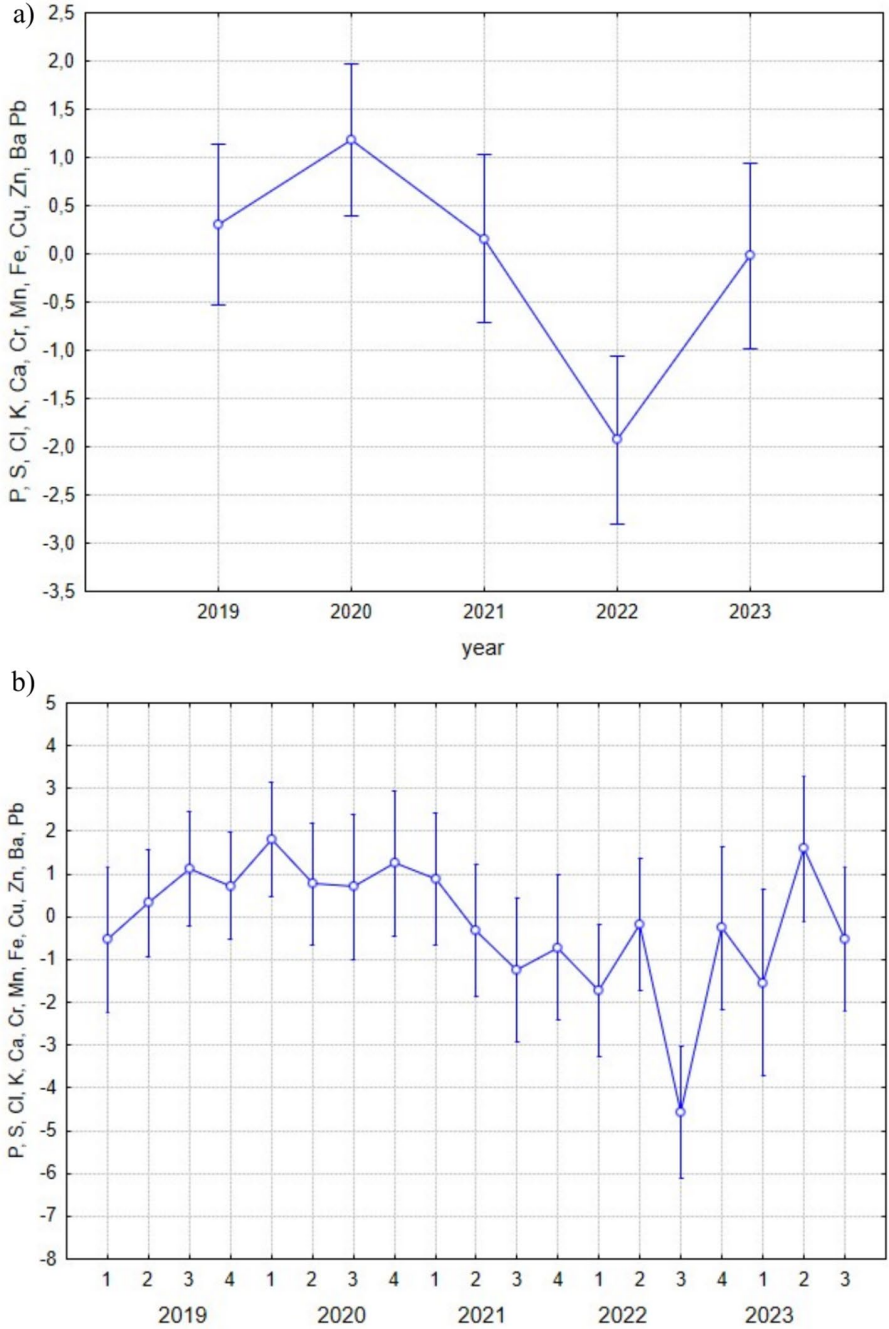
**a** Synergistic accumulation of elements represented by PC1 across years in FFG scrapers (F(4, 107) = 6.4353; *p* = 0.0001). **b** Seasonal variation of PC1 scores within each year (F(18, 93) = 3.0624; *p *= 0.0002). Seasons are coded as: 1—spring, 2—summer, 3—autumn, 4—winter. Data points represent means; error bars indicate ± standard deviation

The total accumulation of elements (PC1) in the bodies of scrapers varied significantly across the study years (F(4, 107) = 6.4353; *p *= 0.0001) (Fig. 3a). The highest levels of accumulated elements were observed in 2022, while the lowest values were recorded in 2020. Seasonal trends in accumulation within individual years were evaluated and found to be significant (F(18, 93) = 3.0624; *p *= 0.0002) (Fig. 3b).

**Fig. 4:**
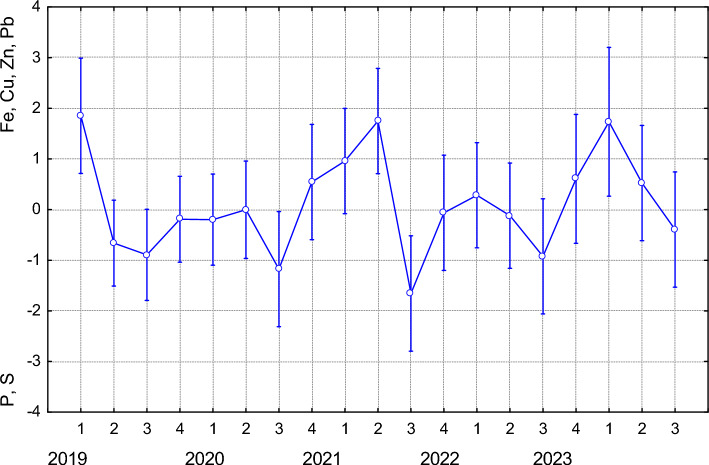
The increase in the accumulation of of Fe, Cu, Zn and Pb, along with simultaneous decrease in the accumulation of P and S (PC2) in FFG scrapers, is significant between seasons within years (F(3, 108) = 7.9457; *p* = 0.0001). In autumn, Fe, Cu, Zn and Pb levels are lowest, while P and S levels are the highest. Seasons are coded as: 1—spring, 2—summer, 3—autumn, 4—winter. Data points represent means; error bars indicate ± standard derivation

The bipolar accumulation of elements represented by PC2 (characterised by increasing levels of Fe, Cu, Zn, and Pb, and decreasing levels of P and S) was not statistically significant between years (F(4, 107) = 1.6812; *p* = 0.1597). However, it varied significantly between seasons within years, with a notable decrease in Fe, Cu, Zn, and Pb, and a concomitant increase in P and S during autumn (Fig. 4).

**Fig. 5:**
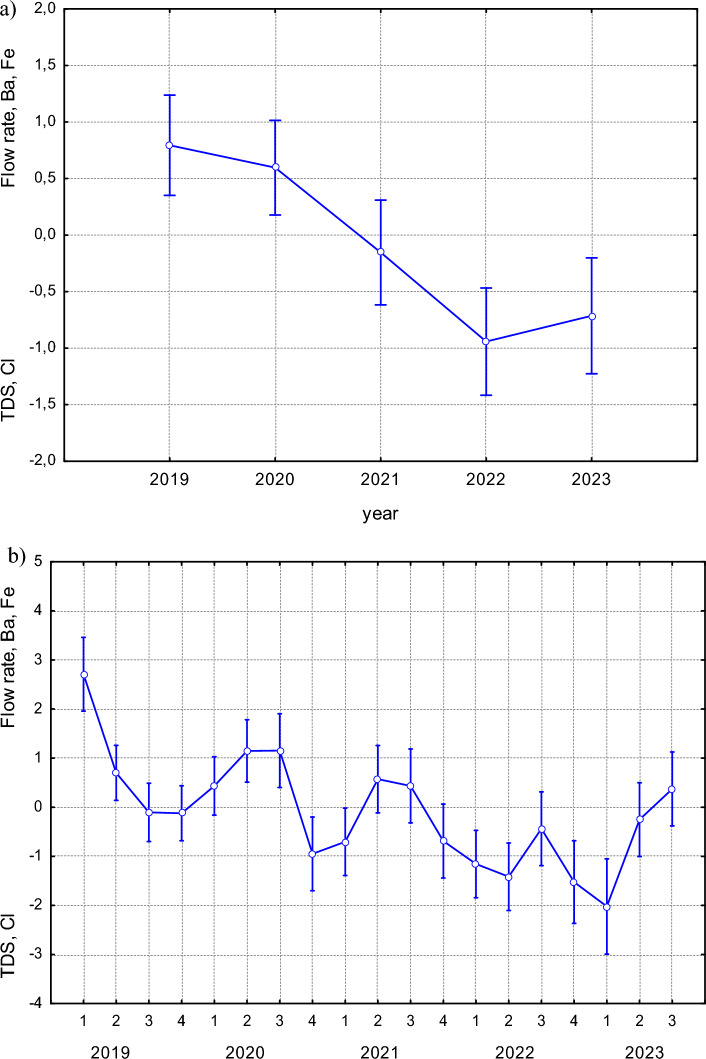
a) Increased accumulation of Fe and Ba with rising flow rates, and a simultaneous decrease in Cl accumulation associated with lower TDS (PC3), differs significantly across years in scrapers (F(4, 107) = 11.034; *p* = 0.0000). b) Seasonal trends in element accumulation related to flow and TDS also show significant variation within each year (F(18, 93) = 8.9102; *p* = 0.0000). Seasons are coded as: 1—spring, 2—summer, 3—autumn, 4—winter. Data points represent means; error bars indicate ± standard deviation of the means

PC3 reflects the accumulation of elements directly associated to water flow rate and TDS. As flow rate increases, the concentrations of Fe and Ba rise, while TDS and Cl decrease. These patterns varied significantly between years. The highest TDS and Cl levels were recorded in 2022, whereas the highest flow rate and concentrations of Fe and Ba were observed in 2019 (Fig. 5a). These values also showed significant seasonal variation across years (Fig. 5b). In 2023, the lowest flow rates and Fe and Ba levels were recorded during winter, while in spring of the same year, these values remained at their lowest.

### Element Accumulation in FFG Predators by Multivariate Comparison

**Fig. 6:**
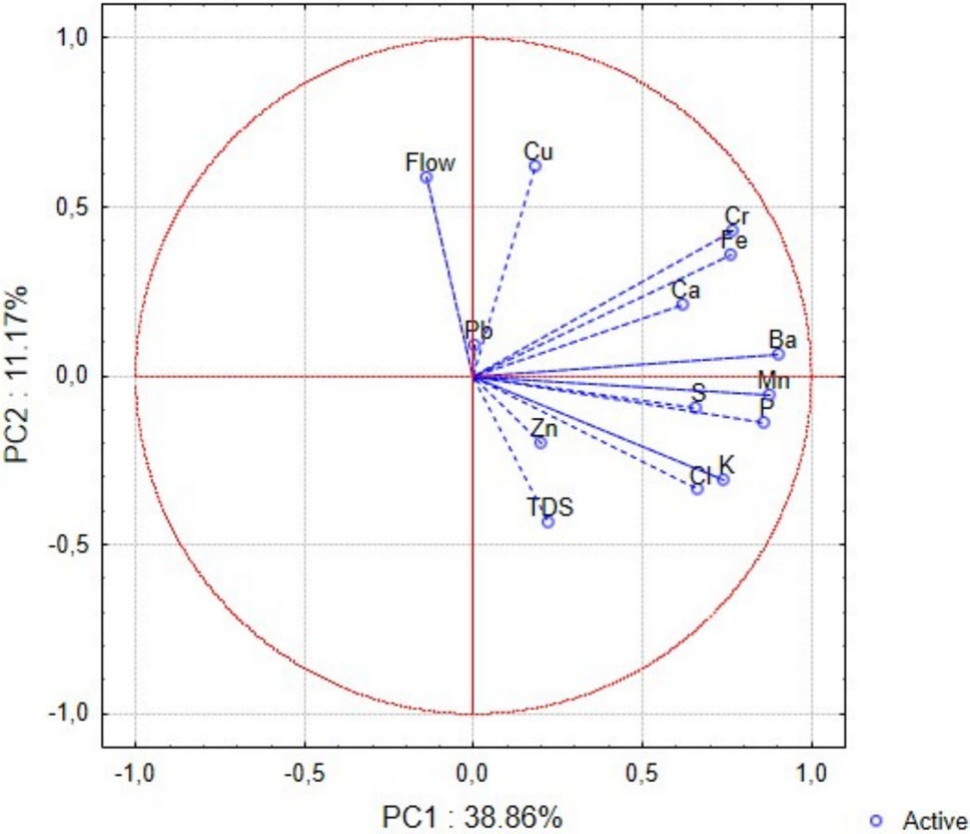
Biplot of principal component analysis (PCA) for FFG predators, illustrating the relationships among environmental variables based on the first two principal components. PC1 explains 38.86% and PC2 explains 11.17% of the total variance. Vectors represent the direction and strength of each variable's contribution to the principal components. Variables such as Flow, TDS, and various elements (e.g., Cu, Cr, Fe, Ca, Zn, Pb) are projected within a unit circle, indicating their correlation structure. Blue circles denote active data points included in the analysis

In the case of FFG predators, the first three PCs are equally important, together explaining more than 60% of the variability in the data (Table 4, Fig. 6). PC1 represents the common accumulation of elements (P, S, Cl, K, Ca, Cr, Mn, Fe, and Ba). Although the number of contributing elements is lower than in the scraper group, a similar trend of simultaneous increase or decrease in accumulation is observed. PC2 reflects the joint accumulation of Cr and Cu, which increases with water flow rate, but TDS decreases. PC3 is also a bipolar factor, representing an increase in S levels with an increasing flow rate and a simultaneous decrease in Zn.

**Table 4:** PC coordinates of variables with the percentage of variation in the PCA of the most frequently and consistently detected elements in the bodies of invertebrate FFG predators, water flow rates, and TDS

	PC1	PC2	PC3
P	**0.862505**	− 0.139221	0.011246
S	**0.657980**	− 0.097482	**0.447387**
Cl	**0.661541**	− 0.338067	0.303702
K	**0.738362**	− 0.309886	0.345359
Ca	**0.617597**	0.207933	− 0.388442
Cr	**0.767792**	**0.429314**	0.025801
Mn	**0.878752**	− 0.054434	− 0.142087
Fe	**0.760901**	0.355951	− 0.174560
Cu	0.181682	**0.617811**	− 0.217942
Zn	0.200515	− 0.200615	− **0.760530**
Ba	**0.904421**	0.065270	0.034076
Pb	0.003073	**0.090834**	− 0.143133
Flow rate	− 0.138203	**0.588448**	**0.424302**
TDS	0.218629	− **0.431706**	− 0.282916
Total variance (%)	38.86	11.17	10.87

The coordinates that contribute the most to the PC value are highlighted in bold

**Fig. 7:**
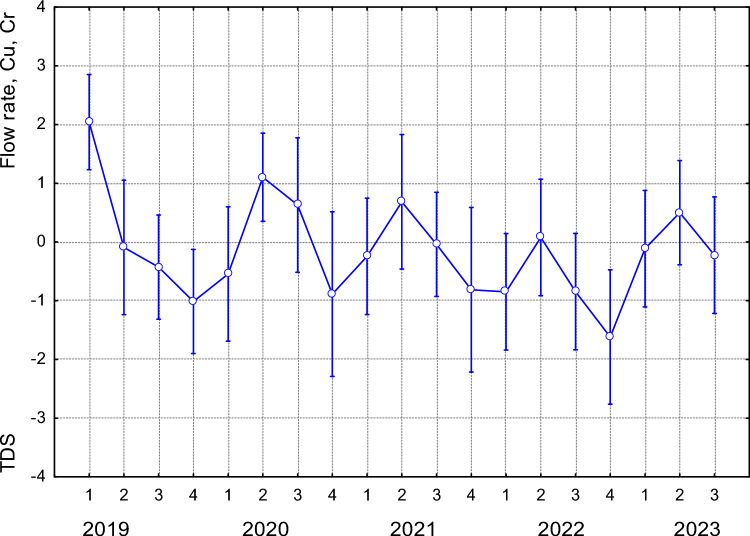
The manifestation of PC2 characterised by increasing levels of Cu and Cr with rising flow rates and decreasing TDS, in FFG predators varied significantly between seasons across the studied years F(18, 57) = 3.4733; *p* = 0.0002). Seasons are coded as: 1—spring, 2—summer, 3—autumn, 4—winter. Data points represent means; error bars indicate ± standard deviation

**Fig. 8:**
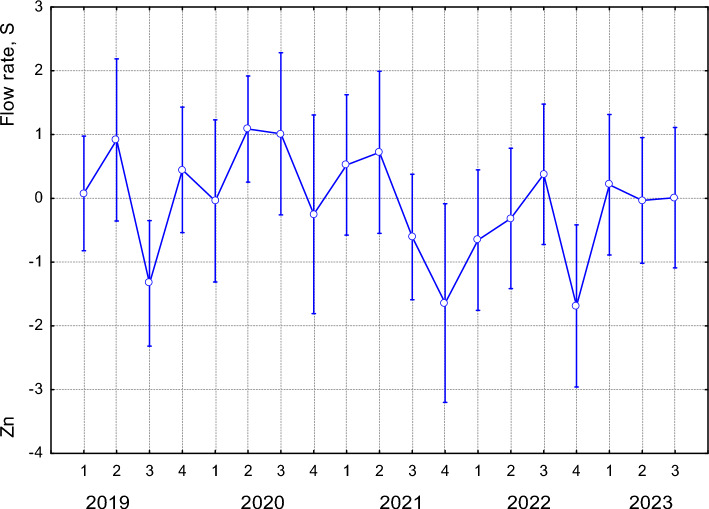
The manifestation of PC3 characterised by increasing water flow rate and S levels, and decreasing Zn levels, varied significantly between seasons across the studied years (F(18, 57) = 2.0773; *p* = 0.0191). Seasons are coded as: 1—spring, 2—summer, 3—autumn, 4—winter. Data points represent means; error bars indicate ± standard deviation

The accumulation of dominant elements on PC1 showed no significant differences between study years in the predator group (F(4, 71) = 1.2271; *p* = 0.3071). Similarly, seasonal variation within years was not statistically significant (F(18, 57) = 1.4002; *p* = 0.1674). PC2, which reflects increasing levels of Cu and Cr with rising flow rates and decreasing TDS, also did not show significant differences between years (F(4, 71) = 2.2069; *p* = 0.08). However, significant seasonal differences were observed across years (Fig. 7), with peaks in spring 2019 and in summer subsequent years. In contrast, the highest TDS values were consistently recorded during winter periods. PC3 represents a bipolar pattern characterised by reduced Zn levels and increased S levels, along with elevated flow rates. As with the previous component, no significant differences were found between years (F(4, 71) = 2.3585; *p* = 0.0616). However, significant differences were observed between seasons within years (Fig. 8). The lowest flow rates and S levels were recorded during winter periods (2020–2022), while in 2019, these values ​​were lowest in autumn.

## Discussion

Aquatic insects are an ecologically important group of aquatic organisms, particularly in lentic and lotic freshwater ecosystems, and thus provide valuable insights into the changing dynamics of aquatic environmnets (Orfinger et al. 2023). Pastorino et al. (2019) organised representative functional feeding groups (FFGs) based on element accumulation and reported a clear separation between them. In our study, the dominant species in the scraper FFG was *Ecdyonurus venosus*, which feeds on diatoms, algae, and plant debris (Lubbock 1866). Algae and diatoms are known for their high capacity to absorb contaminants during bioremediation processes (Roy et al. 2022) and therefore may rapidly reflect changes in elemental concentrations in the aquatic environment (Tuchyňa 2024), suggesting subsequent transfer of elements into the bodies of scrapers. In principal component analysis (PCA), the first principal component typically captures the greatest proportion of variability in the dataset (Jolliffe 2002), allowing the most widespread patterns to be described. In scrapers, principal component (PC)1 reflects high variability across all analysed elements (P, S, Cl, K, Ca, Cr, Mn, Fe, Cu, Zn, Ba, and Pb), whereas in predators, it includes only a subset of elements – primarily those considered biogenically significant, as also noted by Pastorino et al. (2019). PC1 does not correlate with flow rate or TDS in either group. In contrast, PC2 and PC3 are distinct for each FFGs, differ in their associated element groups, and are influenced by flow rate and total dissolved solids (TDS), with patterns varying across years and seasons. These findings indicate that element accumulation patterns are characteristic and specific to each FFG.

### Differences in Element Accumulation Between Years

In the scraper FFG, the content of all analysed elements (PC1) decreased between 2019 and 2020, followed by an increasing trend until 2022, and then declined again in 2023 (Fig. 1a). An opposite trend was observed in PC3, where a decrease in the accumulation of Ba and Fe was associated with reduced water flow, while Cl and TDS increased 2022. In 2023, this trend reversed. In the predator FFG, no such significant interannual trends were observed in the expression of the principal components.

Our results indicate that the concentrations of elements defined by the principal components, which vary significantly between years, follow common trends—either increasing or decreasing until 2022. We assume that this pattern is a consequence of the flash flood that occurred in the Javorinka stream in July 2018. The impact of this event on the physicochemical properties of water (Hrivnáková et al. 2020; Solár et al. 2024a) and on the concentration of certain elements in fish species Alpine bullhead (*Cottus poecilopus*) has already been documented (Janiga et al. 2021). In that study, bullheads exhibited lower concentrations of elements such as Zn, Cr, Rb, Mo, S in their bodies, after the flood compared to the previous year, with further decreases observed in the following year. We propose that the observed increase or decrease in elemental concentrations in aquatic insect larvae is also delayed consequence of this extreme event. Flooding likely caused a reduction in the availability of elements in the stream environment. Their subsequent increase appears to be gradual and long-term, which is reflected in the rising concentrations in biota. Čmelík et al. (2019) reported that heavy metal concentrations (Cd, Cu, Ni, Pb, and Zn) increased during the flood, likely due to sediment disturbance. One year later, concentrations began to return to pre-flood levels, but over time, significant accumulation of heavy metals in sediments was observed. In the heavily polluted river system, pollution levels may recover within 1–2 years over several tens of kilometres (Ciszewski 2001). These findings also support our hypothesis that the increase in elemental concentrations in macrozoobenthos occurs gradually over several years.

**Fig. 9:**
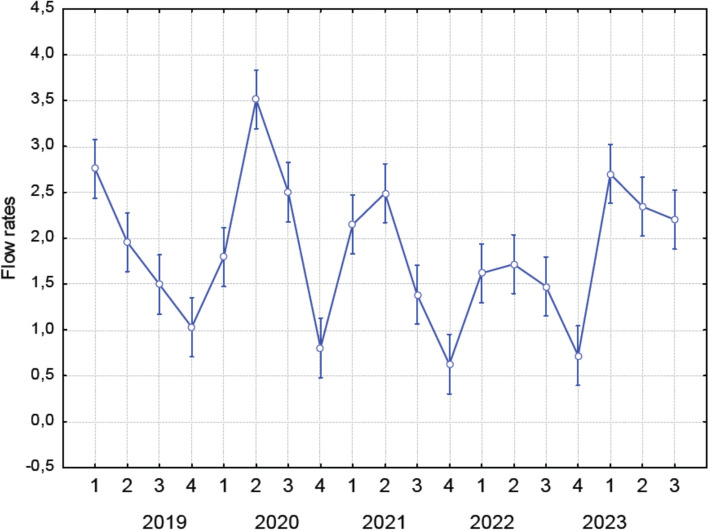
Flow rates in the Javorinka stream across different seasons (F(18, 1717) = 22.098, *p* = 0.0000). Based on average daily flow data provided by the Slovak Hydrometeorological Institute, Košice. 1—spring, 2—summer, 3—autumn, 4—winter. Seasons are coded as: 1—spring, 2—summer, 3—autumn, 4—winter. Data points represent means; error bars indicate ± standard deviation

Based on average flow rate data from the Javorinka stream (Fig. 9), flow rates in 2022 were the lowest recorded during the entire study period. Correspondingly, the highest TDS levels were observed in 2022 (PC3, scrapers FFG). Increased flow rates lead to a decrease in TDS (Dasharath and Reddy 2018), and consequently to reduction in the concentration of elements in the aquatic environment – a pattern that reverses when flow rates are decline. The change in the hydrological regime in 2022 appears to have reversed the previously observed long-term trend of increasing elemental accumulation. Water regime varies from year to year and is influenced by both precipitation and temperature patterns (Cayan et al. 1993). These factors also exhibit seasonal variability, further contributing to fluctuations in flow and elemental dynamics.

### Seasonal Differences in Element Accumulation

In the scraper FFG, PC1-PC3 were significantly associated with seasonal variation. PC1 indicates that the overall accumulation of elements rised until autumn 2022. We hypothesise that seasonal changes in elemental concentrations are primarily driven by fluctuations in stream flow, as our results show the lowest concentrations in summer and the highest in autumn. The accumulation of elements in the aquatic environments depends on various external factors, such as hydraulic loading, tributary water chemistry, and nutrient and sediment input (Reddy et al. 2021). These factors are subject to seasonal variation influenced by climate and weather conditions. The hydrochemical composition of river water also changes seasonally, primarily due to rainfall patterns, which are in turn influenced by the weathering of silicate and carbonate rocks (Thomas et al. 2014). The influx of precipitation into river ecosystem increases the input of elements bound in sediments; however, high flow rates can significantly dilute elemental concentrations. During flood events, certain elements may be radically reduced, and the physicochemical properties of the water can remain altered for several weeks (Janiga et al. 2021). Changes in elemental concentrations in water are transferred through the food chain, and at each trophic level, different factors influence this transfer. Therefore, we hypothesise that, in addition to hydrological conditions, nutritional intake and metabolism of macrozoobenthos represent a second important factor influencing seasonal variation in elemental concentrations.

The spring increase in flow rates in mountain streams is caused by snowmelt at higher elevations during spring and early summer warming. Depending on daily temperatures increase and the extent snow cover, which vary from year to year, the timing of peak flow rates may shift from spring to early summer. Additionally, the amount of precipitation feeding mountain streams typically increases in summer. In winter, precipitation falls as snow, and the streams are often covered by a layer of snow and ice, resulting in the lowest flow rates during this period. Changes in environmental conditions such as temperature, light availability, and water quality can also affect nutrient and element levels. Cooler temperatures in autumn and winter may slow metabolic processes, leading to reduced nutrient uptake (Yao et al. 2021; Skidmore et al. 2023).

The decrease in the levels of Fe, Cu, Zn, and Pb, and the concomitant increase in P and S (PC2, FFG scrapers) is particularly pronounced in autumn period. In autumn, P and S levels are highest, while Zn, Cu, Fe, and Pb levels are lowest. In winter and spring, however, P and S concentrations in insect bodies decrease, and Zn, Cu, Fe, and Pb concentrations increase. These seasonal patterns may reflect changes in food availability and metal bioavailability rather than internal physiological storage, as the organisms analysed were in larval stages. For example, increased runoff and sediment resuspension in spring may enhance the availability of certain metals, while shifts in diet composition or feeding activity may influence the uptake of phosphorus and sulphur. Sulphur and phosphorus share certain chemical properties due to their position in the periodic table (e.g., period 3; five electrons in the p-orbital), which leads to similarities in their chemical behaviour (Scerri 2021), e.g., phosphate and sulphate groups are integral to energy metabolism and introduce negative charges to biological macromolecules (Lima et al. 2022). House and Denison (1998) studied P dynamics in a river and found that seasonal changes affect P transport, with evidence of accumulation during periods of low flow and scouring of bottom sediments during storms. Thus, when P concentrations in water are highest during periods of lowest flow (winter in our case), the accumulation of P in scraper bodies does not necessarily reflect these environmental levels, possibly due to their stronger interaction with sediment-bound phosphorus. Most insects require methionine in their diet, while cysteine can be synthesised from methionine (Jacobsen and Smith 1968; Dadd 1973). With reduced metabolism during winter (Clark and Worland 2008; Rozsypal 2015), there is no uptake of these elements from the diet. Sulphur enters aquatic ecosystems primarily in the form of sulphate, which originates from the weathering of rock minerals in the watershed in the presence of water, as well as from atmospheric sulphur dioxide that reaches the watershed through precipitation and is subsequently transported by surface water system (Luo 2018). In the temperate zones, summer is the season with the highest precipitation and, consequently, the greatest surface runoff (Spaargaren and Deckers 2005). Additionally, higher temperatures can accelerate chemical reactions, leading to faster chemical weathering (Beaulieu et al. 2012; Brantley et al. 2023).

Although Goodyear and McNeill (1998) reported that Ephemeridae (order Ephemeroptera) exhibit weak accumulation relationships with aqueous Pb and Cu, as well as with sediment-bound Zn, our results confirm that the levels of elements Zn, Cu, Fe, and, Pb in the bodies of scrapers increase in spring or summer. This pattern may reflect the environmental availability of elements, potentially released from sediments. High availability of sediment-bound Zn has been observed during periods of low flow (Bambic et al. 2006) or drought (Rue and McKnight 2021). Erel and Morgan (1992) found that elevated levels of anthropogenic Pb, sorbed by Fe oxides, were released during spring snowmelt, whereas in autumn, most natural Pb was released via groundwater flow rather than baseflow.

Another biogenic element associated with this factor is zinc, although its accumulation exhibit the opposite seasonal trend compared to P and S. Zinc functions as a crucial structural, catalytic, and regulatory component of numerous enzymes, transcription factors, and other proteins (Günther et al. 2012). Research by Hare (1992) shows a similar pattern of Zn accumulation in the bodies of *Hexagenia limbata* (Ephemeroptera) as observed in our results. Scrapers accumulated the highest levels of Zn in spring, followed by a decrease in summer and a subsequent increase in autumn. Diet is the primary pathway of Zn bioaccumulation in mayflies, and Zn concentrations in individuals also vary across developmental stages, with subimagos and imagos exhibiting lower Zn levels than larvae (Kim et al. 2012). This finding supports our results, as younger developmental stages were represented in the autumn samples, given that metamorphosis into imago stage occurs during the summer months.

Increases in Fe and Ba levels at elevated flow rates, along with concomitant decreases in Cl and TDS (PC3, scraper FFG), were observed each year, typically reaching their lowest levels in winter. However, in 2023, these values shifted to spring. Increasing Cl concentrations with decreasing flow rates, or conversely, decreasing Cl concentrations due to dilution during periods of high flow caused by precipitation and snowmelt, are supported by the findings of Corsi et al. (2015). Water with low TDS concentrations also promotes Cl leaching from insect bodies, as demonstrated by Noble-Nesbiti (1990). *Sialis* sp. (Sialidae) placed in distilled water continuously lost Cl ions from 0.15–0.35 to 0.1% over five days. However, chlorine can be restored within a few days when larvae are transferred to water containing 1% NaCl. This may suggest that mayflies experience chlorine deficiency in summer during periods of higher flow, as Cl washed out and replenished when flow decreases and TDS increases. Iron becomes more available in water following heavy rainfall or snowmelt, which can transport iron-rich sediments into rivers (Tashiro et al. 2020), consistent with our findings.

In FFG predators, only PC2 and PC3 show significant seasonal differences within the years. In contrast to FFG scrapers, predators have different foraging strategies and ecological roles (Allan et al. 2021). The uptake of nutrients, and thus elements, by predators is determined by the nutrient content of their prey, and animal tissues generally contain nutrients at higher concentrations than plant tissues or detritus (Mendes et al. 2019). Interseasonal differences may result from variations in feeding habits; younger larvae may consume different food types, experience changes of food availability, and undergo physiological changes during ontogeny compared to older larvae or adults (Allan et al. 2021). Elemental uptake may also be influenced by selective feeding within the predator group. The prey consumed by predators can vary in elemental composition, and the waste produced by predators also differs depending on which waste products are preferentially retained or excreted. These processes vary between seasons, highlighting the fundamental role predators play in nutrient cycling within ecosystems (Herzog et al. 2023).

Increasing Cu and Cr levels, together with elevated flow rate and decreased TDS (PC2, FFG predators), peaked in spring 2019 and, in subsequent years, during the summer period. Similar to PC3 in the scraper FFG, we can assume that high summer flows will reduce TDS while simultaneously introducing heavy metals into river through rock weathering caused by snowmelt and precipitation, as well as through channel scouring. Higher flow rates can enhance the mobility of heavy metals such as Cu and Cr. This mobility is positively influenced by rainfall intensity, dissolved oxygen, and water alkalinity, and stream depth, but negatively influenced by pH and stream temperature (Zhang et al. 2024). Our results confirm that increased flows in spring and summer are associated with elevated Cu and Cr levels in bodies of predators; however, other factors reported by Zhang et al. (2024) were not correlated in our study. The transfer of heavy metals bioaccumulated at lower trophic levels may also contribute to increased heavy metal content in predator groups (Solár et al. 2024b). Therefore, during periods of high food resource availability, such as summer, predators may also acquire more Cu and Cr directly from their diet.

Reduced Zn levels and increased S levels, along with elevated flow rates (PC3, FFG predators), were recorded during the winter periods of 2020–2022, whereas in 2019, these values ​​were lowest in autumn. The presence of other metals (e.g., Cd and Mn) may compete with Zn for uptake sites, significantly reduce Zn accumulation in larvae by interfering with same transport systems (Poteat et al. 2012). Therefore, the reduced accumulation of Zn at higher flow rates observed in our results may be explained by the blockage of Zn transport systems by competing elements, particularly during periods of low flow combined with elevated TDS levels. During the spring and summer, i.e., periods of high runoff caused by increased precipitation and snowmelt, the concentration of humic substances in the aquatic environment is typically highest (Vo-Minh Nguyen et al. 2022). The presence of dissolved humic acids in the aquatic environment reduces the toxicity and accumulation of metal ions Zn^2+^ and Cd^2+^ in aquatic insect larvae (Shrestha et al. 2023), which aligns with our findings, where the lowest Zn levels were recorded during the spring and summer seasons. Sulphur uptake in aquatic insect larvae tends to increase during periods when sulphur compounds are more abudant in the environment. In spring and early summer, increased runoff from precipitation and snowmelt can transport more sulphur compounds from decomposing organic matter into the aquatic environment (Jiang et al. 2021). This period often coincides with higher biological activity and larval growth rates (Kivelä et al. 2016), leading to increased S uptake. Our results also confirm elevated S levels in the bodies of FFG predators specifically during the spring and summer seasons.

## Conclusion

We focused on long-term and seasonal trends in the pattern of element accumulation in aquatic insect larval bodies according to their functional feeding group (FFG) affiliation, as the aquatic regime of upland streams is highly dependent on precipitation, which influences the concentration of substances and elements in the aquatic environment. Elements with similar accumulation patterns were grouped in principal component analysis (PCA) to define common principal components (PCs) that varied significantly between years or between seasons within years. Periodic monitoring of elemental accumulation in the FFGs allowed us to assess accumulation patterns over a 5-year period and to identify which elements exhibited seasonal variation, either increasing or decreasing, or whether water flow and TDS influenced differences in accumulation. These trends confirm the differential accumulation of elements based on FFG affiliation.

Scrapers (Ephemeroptera) showed more pronounced interannual variation, with a general increase in element accumulation from 2020 to 2022, likely reflecting post-flood recovery and changes in water chemistry. In contrast, predators (Plecoptera) exhibited more stable accumulation patterns across years, but both groups showed significant seasonal variation in specific elements. Scrapers were more prone to metal accumulation, particularly for Fe, Mn, Zn, and Pb, likely due to their feeding on periphyton and biofilms in direct contact with sediments. Predators accumulated elements such as Cu and Cr primarily through trophic transfer, with seasonal peaks linked to prey availability and hydrological conditions. Seasonal accumulation patterns appear to be closely linked to environmental factors such as stream flow rate, food availability, and physiological changes during ontogeny. For example, Fe, Cu, Zn, and Pb peaked in spring and summer, while P and S were highest in autumn, indicating shifts in bioavailability and dietary intake.

In summary, our findings highlight the importance of feeding strategy and ecological role in shaping element accumulation patterns in aquatic insects. The use of FFG-based approaches provides valuable insights into ecosystem processes and supports their application in long-term biomonitoring of mountain streams.

## Supplementary Information

Below is the link to the electronic supplementary material.

Supplementary file1 (XLSX 38 KB)

## Data Availability

The datasets generated during and/or analyzed during the current study are available from the corresponding author on reasonable request.
